# Oral Manifestations of COVID-19 in Hospitalized Patients: A Systematic Review

**DOI:** 10.3390/ijerph182312511

**Published:** 2021-11-27

**Authors:** Giulia Orilisi, Marco Mascitti, Lucrezia Togni, Riccardo Monterubbianesi, Vincenzo Tosco, Flavia Vitiello, Andrea Santarelli, Angelo Putignano, Giovanna Orsini

**Affiliations:** 1 Department of Clinical, Specialistic and Dental Sciences, Marche Polytechnic University, Via Tronto 10, 60126 Ancona, Italy; g.orilisi@pm.univpm.it (G.O.); m.mascitti@staff.univpm.it (M.M.); togni.lucrezia@gmail.com (L.T.); r.monterubbianesi@univpm.it (R.M.); v.tosco@pm.univpm.it (V.T.); f.vitiello@pm.univpm.it (F.V.); andrea.santarelli@staff.univpm.it (A.S.); a.putignano@univpm.it (A.P.); 2 Dentistry Clinic, National Institute of Health and Science of Aging, IRCCS INRCA, Via Tronto 10, 60126 Ancona, Italy

**Keywords:** SARS-CoV-2, COVID-19, oral lesions, hospitalized patients, oral ulcers, tongue lesions, oral candidiasis

## Abstract

Background: COVID-19 disease first appeared in 2019 and quickly spread worldwide, causing a global pandemic. The oral cavity represents a target of SARS-CoV-2, and oral lesions are observed in both non-hospitalized and hospitalized patients. This systematic review aims to investigate the frequency of oral manifestations in COVID-19 hospitalized patients. Methods: An electronic search was conducted in PubMed, Scopus, and Web of Science databases, including articles published up to September 2021. The review protocol was based on PRISMA-P. The risk of bias of the studies was assessed using the Joana Briggs Institute. The certainty of evidence was assessed using the GRADE instrument. Results: Fifty-nine articles were included: 19 case reports, 17 case series, 2 case-control studies, 13 cross-sectional studies, 4 observational studies, and 4 retrospective studies. Oral ulcers, cheilitis, and tongue lesions were more common in patients before hospitalization, while perioral pressure ulcers, macroglossia, blisters, and oral candidiasis were more recurrent in patients during hospitalization. The first could be related directly to COVID-19, while the latter could be caused by medical devices, treatments, prone position, and immunological impairment. Conclusions: An accurate oral examination during the hospital admission of all confirmed COVID-19 cases is encouraged to recognize oral early manifestations and to apply appropriate treatments.

## 1. Introduction

The outbreak of novel coronavirus SARS-CoV-2 has created a global crisis and challenged healthcare systems across the world [1]. It was first identified in Wuhan, China, on 31 December 2019, in association with a severe human respiratory disease (COVID-19). On 30 January 2020, the World Health Organization (WHO) declared the COVID-19 infection as a Public Health Emergency of International Concern.

SARS-CoV-2 is an enveloped, positive-sense, single-stranded RNA virus with multiple spikes on the surface and a genome size of approximately 26–32 kilobases. Fever, dyspnea, body aches, and dry cough are the most common symptoms [2], but severe cases can develop pneumonia, severe acute respiratory syndrome, and kidney failure, representing a life-threatening condition [3,4]. Most patients display moderate symptoms (80%), while 20% of them may develop a severe disease and 5% may become critically ill, developing pneumonia or acute respiratory distress syndrome, which requires mechanical ventilation and intensive care unit hospitalization [5]. SARS-CoV-2 mainly spreads through respiratory droplets, aerosols, contact, and fomites [6,7,8,9].

A recent study has shown that coronavirus invades human cells via the receptor angiotensin-converting enzyme 2 (ACE2), using the spike-like protein [10]. The ACE2 receptors are located in many organs and tissues, such as skin, olfactory system, and oral cavity; therefore, these cells may host the virus, triggering the inflammatory response [11,12].

Oral manifestations have been reported in several literature studies [13,14,15,16]. The main symptoms, displayed during the pre-symptomatic stage, are ageusia (loss of taste), non-specific anosmia (loss of smell), and hyposalivation [17,18]. Moreover, the most frequently reported oral signs include ulcerative lesions, vesiculobullous/macular lesions, desquamative gingivitis, petechiae, and coinfections such as candidiasis [15,19]. Palate and tongue represent the most involved oral subsites, followed by gingiva and lips [15].

The morphological spectrum of mucocutaneous diseases is still uncertain, as is its relationship with the clinical pattern and course of affected patients. However, many hypotheses have been proposed regarding the etiology of these diseases: direct SARS-CoV-2 infection, a coinfection, a consequence of the impaired immune system, and an adverse reaction to medical treatments and devices [20,21,22]. According to Sarode et al., oral manifestations seen in COVID-19 could be related to SARS-CoV-2 induced anemia [23]. Indeed, ACE2, CD147, and CD26 receptors located on the erythrocytes are potential targets for SARS-CoV-2 attachment, which can lead to hemolysis [24]. On the other hand, some authors have suggested that mucosal ulcers could be considered, not as a primary manifestation of COVID-19, but as a secondary signs related to bacterial and viral coinfections [25]. According to Hocková et al., oral mucocutaneous complications could be caused by the prolonged prone position and mechanical ventilation devices of the intensive care unit (ICU) setting [21]. Most of these studies were case reports and case series; therefore, it is still unclear whether the reported cases were directly due to the COVID-19 infection or indirectly related to stress, anxiety, comorbidities, and medical treatments.

Since, in April 2020, Carreras-Presas et al. published the first work on oral manifestations associated with COVID-19, their prevalence is becoming an emerging concern for the management of these patients [26]. However, few studies have deeply examined this issue. Therefore, the objective of this systematic review was to provide a comprehensive up-to-date summary of the prevalence of oral manifestations in hospitalized patients with COVID-19, reviewing all relevant studies to answer the following question: What are the oral signs and symptoms in hospitalized patients with COVID-19? This systematic review could help the clinicians to focus on detailed intraoral examination of hospitalized patients before and during their admission.

## 2. Materials and Methods

### 2.1. Protocol

The systematic review was performed in accordance with the Preferred Reporting Items for Systematic Reviews and Meta-Analyses (PRISMA), following their checklist [26,27]. The review protocol was based on PRISMA-P [27,28].

### 2.2. Search Strategy

An electronic search was conducted in PubMed, Scopus, and Web of Science for literature updated from 1 January 2020 until 30 September 2021. The following keywords were used in each database: “oral manifestation COVID-19”, “oral manifestation SARS-CoV-2”, “oral manifestation novel coronavirus disease”, “oral lesion COVID-19”, “oral lesion SARS-CoV-2”, and “oral lesion novel coronavirus disease”. Related articles in the reference lists, cited by relevant studies, were manually searched.

### 2.3. Article Selection and Eligibility Criteria

Studies were selected by title and abstract, and full-text screening. Articles were included if they reported oral manifestations in hospitalized patients with COVID-19 infection. 

The full-text articles of all potential studies were evaluated according to the eligibility criteria. The inclusion criteria were: (1) hospitalized patient affected by COVID-19, confirmed through RT-PCR testing; (2) articles reporting oral manifestation associated with COVID-19; and (3) studies published in the English language. The exclusion criteria were: (1) not-confirmed COVID-19 cases; (2) duplicate studies and data; and (3) full-text not accessible. There were no disagreements during the article selection process.

The primary outcome was to highlight the oral signs and symptoms that can occur in hospitalized patients affected by COVID-19. 

**Figure 1:**
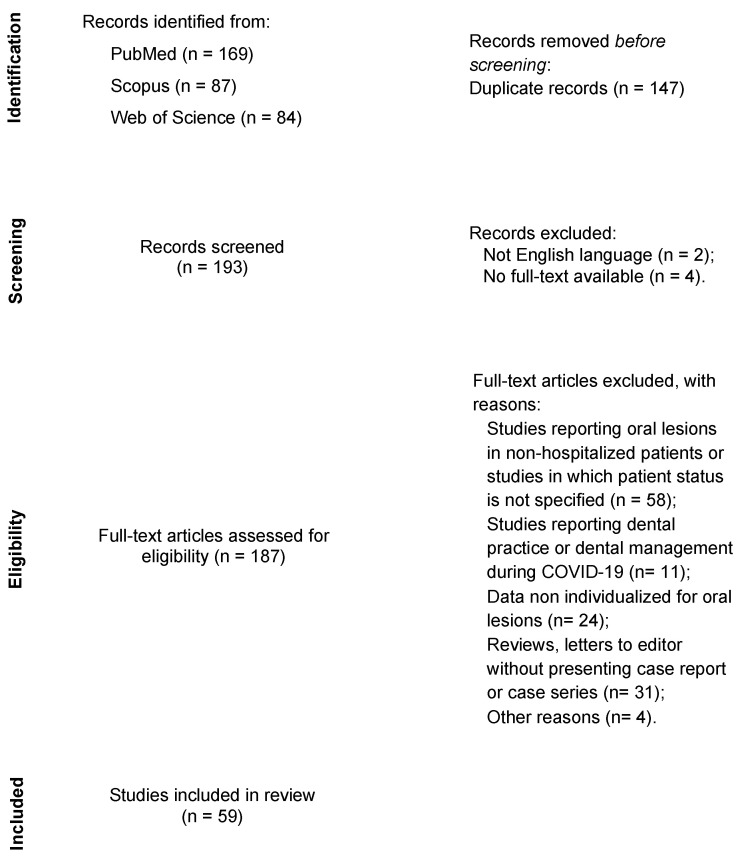
Flow diagram of literature search and selection criteria adapted from PRISMA (Preferred Reporting Items for Systematic Reviews and Meta-Analyses).

A flow diagram detailing the process is presented in Figure 1.

### 2.4. Quality Assessment

The risk of bias of each study was assessed by two blinded reviewers using the Joanna Briggs Institute (JBI) critical appraisal checklist for case reports [29,30]. The score system was agreed by all reviewers before the critical appraisal assessments, and studies were classified according to the following categories: (a) low risk of bias, if the studies reached more than 70% scores of “yes”; (b) moderate risk of bias, if “yes” scores ranged from 50% to 69%; and (c) high risk of bias, if “yes” scores were less than 49%. Data are reported in Supplementary Materials (Table S1).

### 2.5. Certainty of Evidence

The evidence levels of the selected studies were assessed using the GRADE instrument (http://gradepro.org (accessed on 28 October 2021)) [31]. The certainty of evidence was rated for oral lesions prevalence in hospitalized patient. This assessment was based on study design, risk of bias, inconsistency, indirectness, imprecision, and other considerations. Evidence quality was characterized as high, moderate, low, or very low [32]. Data are reported in Supplementary Materials (Table S2).

### 2.6. Statistical Analysis

Descriptive statistical analysis was conducted by grouping and comparing data using Microsoft Excel software (2019, Microsoft Corporation, Redmond, WA, USA)

## 3. Results

### 3.1. Study Selection and Characteristics

A total of 340 studies were identified from databases, and, after removing the duplicate, 193 studies remained for title and abstract screening. A full-text reading was conducted on 187 studies. A total of 128 studies were excluded according to the eligibility criteria. Therefore, 59 studies were selected: 19 case reports, 17 case series, 2 case-control studies, 13 cross-sectional studies, 4 observational studies, and 4 retrospective studies (Table S1). Due to the lack of available data, case reports and series were also included in this systematic review. Thus, the risk-of-bias of each study was evaluated using the JBI critical appraisal checklist [33]. A flowchart detailing the process is presented in Table S1.

A total of 35 articles were judged as low risk [21,26,34,35,36,37,38,39,40,41,42,43,44,45,46,47,48,49,50,51,52,53,54,55,56,57,58,59,60,61,62,63,64,65,66], 12 as a moderate risk [67,68,69,70,71,72,73,74,75,76,77,78], and 12 as high risk [79,80,81,82,83,84,85,86,87,88,89,90] (Table S1).

The sample consisted of 1219 patients, of which there were 456 men (37.4%), 374 women (30.7%), and 389 patients with no specified sex (31.9%), with a mean age of 50.8 years. Results from each selected article were subdivided into “oral lesions appeared before hospital admission” and “oral lesions appeared during hospitalization”. The studies were set in two tables (Table 1 and Table 2), reporting the following data: first author’s name and year of publication, study design, sample size and gender, mean age, medical history, oral manifestations, time of onset, affected site, treatment of COVID-19, treatment of oral manifestation, disease duration, reported diagnosis, and the risk of bias. 

Moreover, data were categorized and described according to the following eight groups: (1) tongue lesions; (2) ulcerative and erosive lesions; (3) aphthous-like lesions; (4) vesiculobullous lesions; (5) lip lesions; (6) functional disorders; (7) candidiasis; and (8) non-specific lesions (mucositis).

### 3.2. Tongue Lesions 

Tongue lesions appearing before hospital admission were reported in 14 studies [37,38,43,51,61,63,65,70,71,74,81,87,88,89], while 14 articles showed that tongue manifestations occurred during hospitalization [21,34,36,39,48,54,59,60,66,69,70,82,84]. The sample consisted of 172 males (age: 57.0 ± 15.1) and 155 females (age: 46.1 ± 22.1). Moreover, 135 patients with no specified sex and mean age were included.

Before hospitalization, white tongue, necrosis of the dorsal tongue, glossitis, geographic and fissured tongue, strawberry tongue, and depapillation of the tongue were the most common lesions diagnosed, directly related to SARS-CoV-2. One patient with a complex medical history showed a 1.5 × 1.5 cm ulcer in the right border of the tongue, while another patient reported a mucopurulent membrane in the anterior dorsal tongue [37]. Only three studies that reported tongue lesions referred to Kawasaki-like disease, potentially associated with COVID-19 [38,51,65].

During hospital admission, the main SARS-CoV-2 related lesions were tongue ulcers, especially in ICU patients [21]. Moreover, white plaque on tongue dorsum, geographic tongue, tongue redness, fissured tongue, and macroglossia were reported [35,54,66]. Macroglossia was reported in two patients who experienced prolonged pronation cycles for several days. Andrews et al. suggested 10 days of methylprednisolone in addition to a bite block to prevent this complication [35]; on the contrary, according to Mascitti et al., macroglossia could be referred to acute lymphatic and vascular obstruction due to COVID-19-related inflammatory response [54].

### 3.3. Ulcerative and Erosive Lesions

Ulcerative and erosive lesions were frequently present in COVID-19 patients, both before and during hospital admission. In particular, 14 studies reported that lesions appeared during hospitalization [21,34,36,37,42,45,47,48,52,57,64,69,70,82], while 6 articles referred to lesions that emerged before hospitalization [36,37,38,41,61,70]. The sample consisted of 111 males (age: 56.7 ± 15.3) and 90 females (age: 53.2 ± 22.4). Moreover, 123 patients with no specified sex and age were included.

Before and during hospitalization, ulcerative and erosive lesions were the most common orofacial manifestations of COVID-19. The ulcers emerged after a latency time of 4 to 7 days after the onset of COVID-19 symptoms, and most of them were diagnosed upon hospital admission. Only in one case did small oral ulcers appear 40 days after a COVID-19 positive test [42]. Some patients displayed painful herpetic and hemorrhagic ulcers with irregular margins, which were variable in size and number. Ulcers that appeared before hospital admission were located on the hard palate and lips, while the lesions that occurred during hospitalization mainly affected the tongue dorsum, lips, and buccal mucosa.

Some authors suggested different factors involved in the development of ulcerative and erosive lesions [12,41,60,61,91]. Ulcers could be related directly to SARS-CoV-2 infection or could be caused by drugs, vasculitis, or thrombotic vasculopathy secondary to COVID-19 [34,41]. Oral ulcerative lesions and erosive plaques appeared a few days after the onset of respiratory symptoms and worsened during hospitalization, due to persistent immunological impairment, and lesions did not heal after SARS-CoV-2 eradication [47]. In four ICU patients, the authors suggested that oral ulcers could be caused by medical devices during the prone positioning phases [21,57]. A wide range of therapies has been used for oral ulcers, including drugs (e.g., dexamethasone, tetracycline) and photobiomodulation therapies [37,57,64].

### 3.4. Aphthous-like Lesions

Aphthous-like lesions were reported in four studies, affecting six minors and three adults (>50 years). Moreover, aphthous-like lesions were showed in 78 cases with various oral signs and symptoms associated with COVID-19 [88]. Lesions were mainly related to COVID-19, probably due to the distribution of the ACE2 receptor on the oral mucosae [11,86], although one patient with minor aphthous ulcers was diagnosed with Sweet syndrome related to COVID-19 [62]. Stress and immunosuppression secondary to COVID-19 infection could be other possible reasons of these lesions [92].

### 3.5. Vesiculobullous Lesions

Vesiculobullous lesions, including herpetiform lesions, angina bullosa-like lesions, and oral blisters, were described in three studies [26,46,53,70]. Carreras-Presas et al. presented a 65-year-old female with lip blisters and desquamative gingivitis, occurring 22 days after viral infection. These lesions seem to be caused by COVID-19 through mechanisms shared with others virus, such as Herpes Simplex Virus-1 (HSV-1)-related gingivostomatitis [26].

Favia et al. detected oral blisters in 19 hospitalized patients (15.4%) that appeared during the first week after the onset of general symptoms and were mainly located on the tongue and palate [70]. According to the authors, lesions could be related to SARS-CoV-2, medical treatments, and/or poor oral hygiene.

Orolabial recurrent herpes simplex were observed in eight patients [46]. This manifestation was found in at HSV-1-positive patients, suggesting a possible superinfection of herpetic virus with COVID-19.

### 3.6. Lip Lesions

Thirteen studies showed patients with lip lesions that appeared before hospital admission [37,38,39,40,41,43,51,63,65,70,71,72,74], and 23 studies during the hospitalization [21,26,37,39,43,45,49,54,56,57,58,59,60,64,66,69,70,73,77,78,82,90]. The reports included 173 males (age: 41.2 ± 25.2), 114 females (age: 52.5 ± 24.3), and 118 patients with no specified sex and age.

Lip lesions included fissured lip, angular cheilitis, and perioral pressure ulcers. Fissured lips have been detected mainly in young patients, who were diagnosed with Kawasaki-like disease related to COVID-19 [39,40,51,54,65,71,72]. Mazzotta et al. described a 9-year-old male with Down syndrome and alopecia areata universalis, presenting glossitis and cheilitis, probably due to the excessive and persistent inflammation that occurred during the interstitial pneumonia with acute respiratory failure [74]. Angular cheilitis, due to stress and immunosuppression, was observed in a 53-year-old man a few days after hospital discharge; after the treatment, this lesion completely disappeared [43].

Prolonged prone position and endotracheal intubation were the most common risk factors for perioral and lip pressure ulcers, interfering with mechanical ventilation equipment in the critical care setting [21,49,56,58,59,66,73,77,78,90]. Most cases were treated with antimicrobial dressing, debridement of necrotic tissue, and paraffin gauze dressing. Finally, some articles reported lip lesions directly related to COVID-19, which appeared as a mucopurulent membrane with superficial necrosis or swollen lips and disappeared after 10–12 days of treatment [37,63,71].

### 3.7. Functional Disorders

Functional disorders included xerostomia, viscous saliva, ageusia and dysgeusia, halitosis, burning mouth, masticatory muscle weakness, salivary gland ectasia, temporo-mandibular article abnormalities, and facial tingling. These disorders were reported by several studies referred to hospitalized patients affected by COVD-19 [3,34,43,76,82,88]. The authors suggested these alterations could be directly related to SARS-CoV-2 and could be considered as an early manifestation of COVID-19 infection. Indeed, ACE2 receptors have been found in the taste buds and glandular epithelium, leading to salivary gland dysfunction and salivary flow impairment [93].

Masticatory muscle weakness, salivary gland ectasia, temporo-mandibular article abnormalities, and facial tingling were reported by Gherlone et al. [48]. The authors suggested that salivary gland ectasia reflected the hyperinflammatory response to SARS-CoV-2, as demonstrated by the significant relationship with C-reactive protein and lactate dehydrogenase levels at hospital admission, and antibiotics use during acute disease.

### 3.8. Candidiasis

Candidiasis appeared as white plaques on the dorsum of tongue, gingiva, and palate [43,44,66,70,75,80,81]. The authors suggested it could be related to the long-lasting antibiotic therapy, the deterioration of general status, and poor oral hygiene [80]. Salehi et al. isolated 65 species of Candida spp. (70.7% *C. albicans*) in a cohort of COVID-19 patients with oropharyngeal candidiasis [75]. Interestingly, Dima et al. reported oral candidiasis with diaper erythema in three newborns positive with COVID-19 [44]. The oral candidiasis in young patients was also reported by Bardellini et al. [81], describing two cases of oral pseudomembranous candidiasis diagnosed at the hospitalization.

### 3.9. Nonspecific Lesions (Mucositis)

Petechiae, macules, erythema, stomatitis, brown pigmentation, mucositis, enanthema, and desquamative gingivitis were reported in many hospitalized patients [26]. Vascular disorders could cause mucositis in the affected patients. Indeed, Cruz-Tapia et al. described a 51-year-old female with diffuse vascular-like purple macule on the left palate and a papule-plaque on the right palate [68]. Riad et al. described 13 patients affected by mucositis; enanthema of the buccal mucosa, palate, and gingiva; and depapillation of the tongue, at hospitalization. These lesions disappeared after 7–14 days of “Magic mouthwash” and paracetamol and could be related directly to COVID-19 infection [6,89]. Only one study described a patient affected by oral lichenoid reaction and a case with oral enanthema, directly due to COVID-19 [54]. Marouf et al. suggested that periodontitis was significantly associated with a higher risk of COVID-19 complications, such as ICU admission, assisted ventilation, and the increased markers levels of COVID-19 worse outcome [55].

## 4. Discussion

A broad spectrum of signs and symptoms were reported in association with COVID-19; however, only a few studies highlighted oral clinical manifestations observed in hospitalized patients. The pathogenesis remains unclear, but some hypotheses have been formulated. Xu et al. showed a higher expression of ACE2 in the oral mucosa, especially on the tongue and in the salivary glands [11]. Thus, the oral cavity might be an anatomical site susceptible to SARS-CoV-2 infection [94]. Consequently, the interaction between SARS-CoV-2 and ACE2 might dysregulate the oral keratinocytes’ function, leading to painful oral ulcers [37]. This mechanism could also be the basis of early manifestations of COVID-19, such as taste alteration and xerostomia [95]. The immune response to infection could activate Langerhans cells and lymphocytes, leading to vasculitis and thrombocytopenia, causing oral lesions related to vascular disorders (e.g., petechiae) [19,41,92,96].

It is still unclear whether oral lesions reflect a direct viral cytopathic effect or represent a consequence of stress, poor oral hygiene, systemic infections, medical treatments, or medical devices used during hospital admission [97]. However, to the best of our knowledge, no attempt has been made to review the available literature regarding oral lesions in hospitalized COVID-19 patients. Therefore, this systematic review is the first to characterizs the patterns of oral lesions that occurred in hospitalized patients affected by COVID-19. As intraoral examination has not yet been considered in the screening of the disease, literature still lacks evidence to better understand the onset of oral manifestations. In this review, the most common oral lesions seen in patients before hospital admission are painful ulcers, cheilitis, and tongue lesions. According to several authors, these lesions are related directly to COVID-19 [36,37,88,89]. Conversely, the most common oral lesions displayed by patients during hospitalization are perioral pressure ulcers, macroglossia, blisters, and oral candidiasis. These lesions may be due to the long-lasting prone position of ICU patients [21,90], increased pressure of the endotracheal tubes [59], prolonged inpatient care [52], persistent immunological impairment [43,47], and medical treatments [80].

The studies reporting that oral manifestations in COVID-19 are very low, leading to a considerable risk of bias. Most of the eligible studies showed a high risk of bias, due to insufficient simple size, poor methods, or inadequate designs. Case reports and case series have profoundly influenced the medical literature and continue to improve the current knowledge [98]. Although the evidence derived from case series and case reports is very low, a strong recommendation for oral lesions of hospitalized patients, affected by COVID-19, can be provide. Moreover, GRADE literature describes five paradigmatic situations in which a strong recommendation can be made based on low quality evidence, such as a condition of life threatening [99].

Considering the results of this systematic review, clinicians should consider the oral lesions affecting the hospitalized patients with COVID-19 to ensure an adequate prevention and therapeutic management. Thus, the importance of the oral examination should be emphasized in hospitalized patients with infectious diseases, to encourage the multidisciplinary management of COVID-19 patient.

## 5. Conclusions

The new SARS-CoV-2 has become a worldwide sanitary emergency. Understanding the manifestations and progression of COVID-19 is the first step in developing an adequate prevention and treatment management. Although the typical symptoms include fever, shortness of breath, and dry cough, oral lesions have also been reported. The association between oral diseases and SARS-CoV-2 infection is still unclear and is currently poorly investigated.

Based on this systematic review, it could be suggest that: (1) painful oral ulcers, cheilitis, and tongue lesions are more frequent in patients before hospital admission; (2) perioral pressure ulcers, macroglossia, blisters, and oral candidiasis are more evident in patients during hospitalization; (3) lesions that appeared before hospital admission are mainly related directly to COVID-19; (4) lesions that appeared during hospitalization are mainly associated with medical devices and treatments, prone position, and immunological impairment; (5) all clinicians, during the hospital admission, should be encouraged to perform an accurate oral examination of all confirmed COVID-19 cases to recognize the disease’s possible early manifestations; (6) further studies are necessary to establish the pathological significance of oral manifestations during COVID-19.

## Figures and Tables

**Table 1:** Descriptive characteristics of included studies regarding oral lesions that appeared before hospitalization in patients affected by COVID-19. F: female; M: male; CS: cases series; RS: retrospective study; CSS: cross-sectional study; CR: case report; OS: observational study; NA: not available; COPD: chronic obstructive pulmonary disease; AZT: azithromycin; CRST: corticosteroids; IVIG: Intravenous immunoglobulin; PDT: Photodynamic therapy; PBMT: Photobiomodulation therapy; HCH: hydroxychloroquine; ASA: acetylsalicylic acid.

Study	Design	Sample (n, Sex)	Mean Age (Year)	Medical History	Oral Manifestations	Time of Onset	Affected Site	Treatment COVID-19	Treatment Oral Manifestations	Duration (Days)	Reported Diagnosis	Risk of Bias
Ansari et al., 2021 [36]	CS	1, F	56	Diabetes	Painful ulcers	5 days after COVID-19 symptoms	Hard palate	Remdesivir, AZT	Diphenhydramine, CRST, tetracycline, and lidocaine	7	Oral lesions due to COVID-19	Low
Bardellini et al., 2021 [81]	RS	19, M 8, F	4.2 1.7	NA	Hyperemic pharynx (*n*. 10), Pseudomembranous candidiasis (*n*. 2), Coated tongue (*n*. 2), Geographic tongue (*n*. 1)	Hospital admission	Tongue, pharynx, Oral mucosa	NA	NA	NA	Oral lesions due to COVID-19	High
Brandão et al., 2021 [37]	CS	1, M 2, F	81 77	Hypertension, COPD, Diabetes, Obesity, Pancreatitis	Mucopurulent membrane, Ulcers, Petechia, Necrosis	Hospital admission	Upper and lower lips, Anterior dorsal tongue	AZT, Piperacillin/Tazobactam, Ceftriaxone	Acyclovir, PBMT	5–11	Oral lesions due to COVID-19	Low
Chen et al., 2020 [67]	CSS	15, M 16, F	60.6	NA	Dry mouth	NA	Oral mucosa	NA	NA	NA	Poor oral hygiene or microbiota imbalance due to drugs	Moderate
Chérif et al., 2020 [38]	CR	1, F	35	NA	Ulcers, Enanthem	Hospital admission	Tongue, Lip	NA	HCH, AZT, Cefuroxime	10	Kawasaki-like due to COVID-19	Low
Chiotos et al., 2020 [39]	CS	1, M 1, F	12 5	NA	Fissured lip	Hospital admission	Lip	IVIG, CRST, Milrinone	NA	NA	Kawasaki-like due to COVID-19	Low
Chiu et al., 2020 [40]	CR	1, M	10	NA	Cracked lip, Erythema	Hospital admission	Lip, Oropharynx	Dopamine	NA	NA	Kawasaki-like due to COVID-19	Low
Ciccarese et al, 2021 [41]	CR	1, F	19	NA	Erosions, Ulcers, Petechiae	2 days before hospital admission	Palate, Lips	Cefixime, IVIG, CRST	NA	10	Petechiae due to thrombocytopenia	Low
Cruz-Tapia et al., 2020 [68]	CS	1, F	51	NA	Vascular-like purple macule	Hospital admission	Palate	CRST, Azithromycin, Indomethacin	NA	6	Vascular disorder due to COVID-19	Moderate
Díaz Rodríguez et al., 2020 [43]	CS	1, F	78	NA	Dry mouth, Pseudomembranous candidiasis	Hospital admission	Tongue, Hard and soft palate, Lip	NA	CRST, Neomycin, Mouthwash, Nystatin solution, triamcinolone acetonide	After treatment	Stress and immunosoppression	Low
Dima et al., 2020 [44]	CS	2, M 1, F	14.3 days	NA	Oral candidiasis	Hospital admission (2 weeks after birth)	NA	Vitamin D	Nystatin	NA	NA	Low
Favia et al., 2021 [70]	CSS	70, M 53, F	72	NA	Geographic tongue, Fissured tongue, Ulcers, Blisters, Hyperplasia of papillae, Angina bullosa, Candidiasis, Ulceronecrotic gingivitis, Petechiae	Onset of COVID-19 symptoms; within or after 1 week of COVID-19 symptoms	Tongue, Palate, Lip, Cheek	NA	Hyaluronic acid gel, chlorhexidine, Miconazole, Nitrate Tranexamic acid	NA	Oral lesions due to COVID-19 and poor oral hygiene	Moderate
Halepas et al., 2021 [71]	CSS	23	21	NA	Swollen lips, Strawberry tongue	Hospital admission	Lip, Tongue	NA	NA	NA	Oral lesions due to COVID-19	Moderate
Jones et al., 2020 [51]	CR	1, F	0.5	NA	Cracked lip, Tongue prominent papilla	NA	Lip, Tongue	IVIG, ASA	NA	NA	Kawasaki-like due to COVID-19	Low
Katz et al., 2021 [86]	CR	6, F	NA	NA	Oral aphthae	NA	NA	NA	NA	NA	Oral lesions due to COVID-19	High
Labè et al., 2020 [72]	CS	1, M	6	NA	Erosive cheilitis, Diffuse gingival erosions	Hospital admission	Lip, Gingiva	NA	NA	14	Kawasaki like disease due to COVID-19	Moderate
Mazzotta et al., 2020 [74]	CR	1, M	9	Down syndrome, Alopecia areata universalis	Glossitis, Cheilitis	3 weeks after COVID-19 symptoms	Lip, Tongue	CRST	NA	NA	Oral lesions due to COVID-19	Moderate
McGoldrick et al., 2021 [87]	CS	1, M	53	NA	Swelling	1 day before hospital admission	Tongue, Floor of mouth	CRST	NA	NA	Oral lesions due to COVID-19	High
Nuno-Gonzalez et al. 2021 [88]	CSS	78	NA	NA	Lingual papillitis, Glossitis, Aphthous-like lesions, Patchy depapillation, Mucositis, Burning sensation, Dysgeusia	NA	Tongue, Oral mucosa	NA	NA	NA	Oral lesions due to COVID-19	High
Riad A et al., 2020 [89]	CS	5, M 8, F	51.08	Hypertension, Diabetes, Asthma	Mucositis, Enanthema, Tongue depapillation	0–2 days after COVID-19 symptoms	Palate, Tongue, Buccal mucosa, Gingiva	Paracetamol (*n*. 9), CRST (*n*. 2), Chloroquine (*n*. 2)	Mouthwash and paracetamol	7–14	Oral lesions due to COVID-19	High
Salehi et al., 2020 [75]	CSS	23, M 30, F	27–90	Cardiovascular diseases (*n*. 28), Diabetes (*n*. 20), Chronic kidney diseases (*n*. 11), Hematological malignancies (*n*. 5)	Oropharyngeal candidiasis	1–30 days after COVID−19 symptoms	Oral mucosa	Broad-spectrum antibiotics (*n*. 49), CRST (*n*. 25)	Fluconazole (*n*. 21), Fluconazole and Nystatin (*n*. 13), Nystatin (*n*. 13)	NA	Oral lesions due to COVID-19	Moderate
Soares et al., 2020 [61]	CR	1, M	42	Diabetes, Hypertension	Ulcers, Reddish macules	NA	Lip, Buccal mucosa, Hard palate, Tongue	CRST; Dipyrone	NA	21	Oral lesions due to COVID-19	Low
Taşkın et al., 2020 [62]	CR	1, F	61	NA	Minor aphthous ulcer	Hospital admission	Hard palate, Buccal mucosa	AZT, HCH, Oseltamivir Tocilizomab, Favipiravir	NA	NA	Oral lesions due to COVID-19	Low
Taşlıdere et al., 2021 [63]	CR	1, F	51	Melkersson– Rosenthal syndrome	Swollen lip, Fissured tongue	Hospital admission	Lip, Tongue	AZT, HCH, CRST	NA	NA	Oral lesions due to COVID-19	Low
Verdoni et al., 2020 [65]	OS	2, M	4.5	Kawasaki-like disease	Erosive cheilitis, Diffuse gingival erosions, Glossitis	1 week before/during hospital admission	Lip, Tongue	IVIG, ASA, CRST	NA	NA	Kawasaki like disease due to COVID-19	Low

**Table 2:** Descriptive characteristics of included studies regarding oral lesions that appeared during hospitalization in patients affected by COVID-19. F: female; M: male; CS: cases series; RS: retrospective study; CSS: cross-sectional study; CR: case report; OS: observational study; NA: not available; AZT: azithromycin; CRST: corticosteroids; IVIG: Intravenous immunoglobulin; PDT: Photodynamic therapy; PBMT: Photobiomodulation therapy; HCH: hydroxychloroquine; ASA: acetylsalicylic acid; ICU: intensive care unit.

Study	Design	Sample (n, Sex)	Mean Age (Year)	Medical History	ICU	Oral Manifestations	Time of Onset	Affected Site	Treatment COVID-19	Treatment Oral Manifestations	Duration (Days)	Reported Diagnosis	Risk of Bias
Amorim Dos Santos et al., 2020 [34]	CR	1, M	67	Coronary disease, Hypertension, Autosomal dominant polycystic kidney disease, Kidney transplant	ICU + Orotracheal intubation	White plaque, Multiple pinpoint yellowish ulcers, Geographic and fissured tongue, Viscous saliva	24 days after COVID-19 symptoms	Tongue	Initially: AZT, HCH, Ceftriaxone Later: Meropenem, Sulfamethoxazole, Trimethoprim, Immunosuppressants, Anticoagulants	Fluconazole, Nystatin, Chlorhexidine digluconate, mouth rinses, Hydrogen peroxide	15 (tongue lesions) 17 (geographic tongue)	Oral lesions due to COVID-19	Low
Andrews et al., 2020. [35]	CR	1, F	40	Diabetes, Pancreatic insufficiency, Asthma, Hypertension, Ulcerative colitis	ICU + Tracheostomy	Acute macroglossia	11 days after prone position	Tongue	Initially: HCH, CRST, Tocilizumab. Later: Prone position	CRST, Bite block	11	Oral lesions due to prone position	Low
Ansari et al., 2021 [36]	CS	1, M	75	Hypertension	Hospital admission	Painful ulcers	1 week after hospitalization	Anterior tongue	AZT	CRST, Diphenhydramine, Tetracycline, Lidocaine	7	Oral lesions due to COVID-19	Low
Askin et al., 2020 [79]	OS	123, M 87, F	57.44(M) 58.80(F)	NA	Hospital admission (*n*. 163), ICU (*n*. 47)	Necrosis (*n*. 4), Enanthem, Aphthous stomatitis (*n*. 2)	NA	Maxillary region, Oral mucosa	NA	NA	NA	Oral lesions due to COVID-19 and medical treatments	High
Baraboutis et al., 2020 [80]	CSS	49	NA	NA	Hospital admission	Oral candidiasis	5 days after antimicrobial therapy	NA	HCH (*n*. 46), AZT (*n*. 48)	CRST	NA	Oral lesions due to medical treatments	High
Brandão et al., 2021 [37]	CS	1, M	72	Diabetes, Hypertension	30 days of hospitalization + ICU	Hemorrhagic ulcerations	A few days after hospital admission	Upper and lower lip	Piperacillin/Tazobactam, AZT, Ceftriaxone	Acyclovir, PBMT	7	Oral lesions due to COVID-19	Low
Chiotos et al., 2020 [39]	CS	1, F	9	NA	ICU	Fissured lip, Strawberry tongue	5 days after hospital admission	Lip, Tongue	IVIG, CS, ASA, Milrinone	NA	NA	Kawasaki-like due to COVID-19	Low
De Medeiros et al., 2021 [42]	CR	1, M	43	Hodgkin’s lymphoma	Hospital admission	Ulcers	40 days after COVID-19 diagnosis	NA	CRST; Methotrexate	NA	NA	Oral lesions due to COVID-19	Low
Díaz Rodríguez et al., 2020 [43]	CS	1, M	53	NA	Hospital admission	Burning mouth sensation, Commissural fissures	A few days after hospital discharged	Lip, Mouth	NA	Nystatin, CRST Neomycin, Mouthwash	After treatment	Oral lesions due to immunosuppression	Low
El Kady et al., 2021 [82]	CSS	31, M 27, F	18–46	NA	Hospital admission	Oral ulcers; tongue redness, gingival bleeding and burning sensation	NA	Lip, Tongue, Gingiva	NA	NA	NA	NA	High
Emelyanova et al., 2021 [69]	CR	1, M	38	NA	Hospital admission	Redness and scale-crusts lips, Keratosis, Desquamations	5 days after COVID-19 symptoms	Lip, Tongue	NA	CRST, Levaxela, Clexane, Vitamin C-D, Zinc, Famotidine	NA	NA	Moderate
Fathi et al., 2021 [45]	CR	1, F	22	NA	ICU	Ulcers	Three days after ICU	Lip, Mouth	Metronidazole, Ceftriaxone, Meropenem, Ribavirin, HCH	Oral valaciclovir	NA	Oral lesions due to COVID-19	Low
Favia et al., 2021 [70]	CSS	70, M 53, F	72	NA	Hospital admission + ICU	Geographic and fissured tongue, Ulcers, Blisters, Hyperplasia of papillae, Angina bulllosa, Candidiasis, ulcero-necrotic Gingivitis, Petechiae	Onset of COVID-19 symptoms; within 1 week after COVID-19 symptoms; after 1 week of COVID-19 symptoms	Tongue, Palate, Lip, Cheek	NA	Hyaluronic acid gel, Chlorhexidine, Miconazole, Nitrate Tranexamic acid	NA	Oral lesions due to COVID-19 and poor oral hygiene	Moderate
Fernandez-Nieto et al., 2020 [46]	CSS	5, M 3, F	61.8 72.7	Hypertension, Chronic kidney disease, Hyperuricemia, Dyslipidemia, Colorectal cancer	Hospital admission; ICU	Herpes simplex	NA	Lip	HCH, AZT, ceftriaxone, acyclovir	NA	NA	Herpetic infections and superinfections in patients with COVID-19	Low
Gabusi et al., 2021 [47]	CR	1	78	NA	Hospital admission	Ulcers, Erosive plaques	A few days after COVID-19 symptoms	NA	HCH, CRST, Ciprofloxacin, Tocilizumab	CRST, Chlorhexidine gel, and topical lidocaine	NA	Persistent immunological impairment	Low
Gherlone et al., 2021 [48]	CSS	122	62.5	NA	Hospital admission + ICU	Salivary gland ectasia (*n*. 46), TMJ alterations (*n*. 9), Masticatory muscle weakness, Oral ulcers, Dry mouth, Facial tingling, White tongue.	NA	Salivary gland, TMJ, Oral mucosae, Facial tissues, Tongue	NA	NA	NA	Oral lesions due to COVID-19	Low
Hedou et al., 2020 [83]	CSS	4	47	NA	ICU	Erythematous rash	NA	NA	NA	NA	NA	NA	High
Hocková et al., 2021 [21]	CS	3, M	64.3	Hypertension, chronic hepatopathy, Hypercholesterolemia, Gastroesophageal reflux disease, Obesity,	ICU	Haemorrhagic ulcers, Acute bilateral parotitis	After ICU admission	Tongue, Lip, Parotid	Ceftriaxone, Clarithromycin, Remdesivir, Paracetamol, CRST, Vitamin C and B1, Nadroparin, Inosine pranobex, Atorvastatin, Lagosa, Vitamin D, Zinc, Famotidine	Dressing, position Adjustment, antifungals, antivirals, Surgical interventions	7–14	Oral lesions due to medical devices	Low
Horzov et al., 2021 [84]	RS	64, M 71, F	48.7	NA	Hospital admission	Tongue plaque	NA	Tongue	NA	NA	NA	Oral lesions due to COVID-19	High
Ibarra et al., 2021 [49]	CCS	41, M 16, F	61	NA	ICU	Perioral pressure ulcers	During ICU	Perioral tissues	Prone position	Dressing	NA	Prone position	Low
Jiménez-Cahué et al., 2020 [50]	CS	3, F	63	NA	Hospital admission	Macules, Petechiae	24 days after COVID-19 symptoms	Palate	lopinavir, HCH, AZT, CRST, Ceftriaxone	AZT, Ceftriaxone, CRST, HCH	NA	Oral lesions due to COVID-19	Low
Jiménez-Cauhè et al., 2020 [85]	CSS	6	40–69	NA	ICU	Enanthema	2–24 days from COVID-19 symptoms	Palate	NA	NA	NA	NA	High
Kämmerer et al., 2020 [52]	CR	1, M	46	Hypercholesterinemia, Coronary heart disease	Hospital admission + ICU	Herpetic ulcers	3 days after intubation	Oral cavity, Gingiva	Aciclovir	NA	NA	Herpetic infections and superinfections in patients with COVID-19	Low
Llamas-Velasco et al., 2020 [53]	CS	1, F	59	NA	ICU	Vesicles	25 days after COVID-19 symptoms	Perioral tissues	HCH, Lopinavir/Ritonavir, Ceftriaxone	NA	NA	Oral lesions due to COVID-19	Low
Martel and Orgill, 2020 [73]	CS	18	NA	NA	ICU	Perioral pressure ulcers	During ICU	Perioral tissues	NA	Dressing	NA	Prone position and medical devices	Moderate
Carreras-Presas et al., 2021 [26]	CS	1, F	65	Obesity, Hypertension	Hospital admission	Blisters, Desquamative gingivitis	22 days after COVID-19 symptoms; 4 days after hospital discharged	Gingiva, Lip	Lopinavir, Ritonavir, HCH	Hyaluronic acid and chlorhexidine mouthwash, CRST	3	Oral lesions due to COVID-19	Low
Mascitti et al., 2021 [54]	CSS	39	NA	NA	Hospital admission	Oral lichenoid reaction, Enanthema, Macroglossia, Cheilitis	NA	Oral mucosae, Tongue, Lip	Antibiotics, AZT, HCH	NA	NA	Oral lesions due to COVID-19	Low
Marouf et al., 2021 [55]	CCS	20, M 20, F	53.6	NA	Hospital admission + ICU	Periodontitis	NA	Periodontium	NA	NA	NA	NA	Low
Perrillat et al., 2020 [56]	CS	2, M	38.5	Obesity	ICU	Perioral pressure ulcers	After ICU admission	Perioral tissues	Prone position	Dressing	NA	Prone position	Low
Ramires et al., 2021 [57]	CR	1, F	50	Obesity, Hypertension, Diabetes	ICU	Ulcers	4 days after extubation	Lip	NA	PBMT, PDT	4	Prone position	Low
Ramondetta et al., 2020 [58]	CR	1, M	48	NA	ICU	Perioral pressure ulcers	15 days after ICU admission	Perioral tissues	Initially: HCH, antivirals Later: prone position	Dressing	NA	Prone position	Low
Rekhtman et al., 2021 [59]	CSS	3	NA	NA	ICU	Perioral pressure ulcers	NA	Lip, Tongue	Mechanical ventilation	NA	NA	Oral lesions due to medical devices	Low
Shearer et al., 2021 [90]	RS	68	61.3	NA	Hospital admission + ICU	Perioral pressure ulcers	NA	Perioral tissues	Mechanical ventilation, Prone positioning, Endotracheal intubation	Dressing	NA	Prone position	High
Singh et al., 2020 [60]	CS	1, M 1, F	44 71	Diabetes, Hypertension	Hospital admission + ICU	Extensive mucosal damage, Discolorations of lip and tongue	4/5 days after prone positioning	Lip, Tongue	AZT, CRST, Mechanical ventilation	Prone position, dressing	NA	Prone position	Low
Sinjari et al., 2020 [76]	OS	20	69.2	Hypertension, Heart, Respiratory and Thyroid disease, Cancer, Diabetes	Hospital admission	Dysgeusia (*n*. 5), Burning sensation (*n*. 3), Dysphagia (*n*. 4)	NA	Mouth	NA	NA	NA	Oral lesions due to COVID-19	Moderate
Siotos et al., 2020 [77]	CR	1, F	82	Hypertension, Hyperlipidemia	ICU	Perioral pressure ulcers	10 days after prone positioning	Perioral tissues	Mechanical ventilation, Prone position	Dressing	NA	Prone position	Moderate
Sleiwah et al., 2020 [78]	RS	14, M 2, F	58.6	NA	ICU	Perioral pressure ulcers	NA	Perioral tissues	Mechanical ventilation, Prone position	NA	NA	Prone position	Moderate
Teixeira et al., 2021 [64]	CS	1, M 3, F	57 72.7	Hypertension, Hypothyroidism, Rectal tumor	Hospital admission, ICU	NA	10 days after hospital readmission; after ICU admission	Lip	AZT, Piperacillin and Tazobactam, Ceftraxone/Cefuroxime, Ivermectin	PBMT, PDT	1–4	Oral lesions due to COVID-19	Low
Zingarelli et al., 2020 [66]	CR	1, F	50	NA	ICU	Perioral pressure ulcers, Candidiasis, Stomatitis, Macroglossia	15 days after ICU admission	Perioral tissues, Tongue	Mechanical ventilation	Dressing	7	Prone position	Low

## Data Availability

The data presented in this study are available on request from the corresponding author.

## Supplementary Materials

The following are available online at https://www.mdpi.com/article/10.3390/ijerph182312511/s1, Table S1: Risk of bias in the reported articles, Table S2: Certainty assessment and grading of the evidence.
