# Supplementary material for: Oral Manifestations of COVID-19 in Hospitalized Patients: A Systematic Review
Source: Int J Environ Res Public Health. 2021 Nov 27;18(23):12511. doi: 10.3390/ijerph182312511 (PMC8656958; doi:10.3390/ijerph182312511)
Supplement: Supplementary file 1 [file ijerph-18-12511-s001.zip › ijerph-1472538-supplementary.pdf]

SUPPLEMENTARY MATERIALS

Table S1. *Risk of bias in the reported articles*. Study characteristics and risk of bias assessed by the Joanna Briggs Institute critical appraisal tool for case reports and, level of evidence and grade of recommendation of Oxford Centre for Evidence-Based Medicine.
Q1. Were patient's demographic characteristics clearly described?
Q2. Was the patient's history clearly described and presented as a timeline?
Q3. Was the current clinical condition of the patient on presentation clearly described?
Q4. Were diagnostic tests or assessment methods and the results clearly described?
Q5. Was the intervention(s) or treatment procedure(s) clearly described?
Q6. Was the post-intervention clinical condition clearly described?
Q7. Were adverse events (harms) or unanticipated events identified and described?
Q8. Does the case report provide takeaway lessons?
Abbreviations: Y: Yes; N: No; NA: Not applicable; U: Unclear; CCS: Case-control study; CR: Case report; CS: Case series; CSS: Cross-sectional study; OS: Observational study; RS: Retrospective study.

		**Joana Riggs Institute check list**								
**Authors**	**Type of study**	**Q1**	**Q2**	**Q3**	**Q4**	**Q5**	**Q6**	**Q7**	**Q8**	**Total score/ Risk of bias**
Amorim Dos Santos et al., 2020 [34]	CR	Y	Y	Y	Y	Y	Y	NA	Y	100%, low
Andrews et al., 2020 [35]	CR	Y	Y	Y	Y	Y	Y	NA	Y	100%, low
Ansari et al. 2021 [36]	CS	Y	Y	N	Y	Y	Y	NA	Y	85%, low
Askin et al., 2020 [79]	OS	Y	N	N	Y	N	N	NA	Y	43%, high
Baraboutis et al., 2020 [80]	CSS	Y	N	N	Y	N	N	NA	Y	43%, high
Bardellini et al., 2021 [81]	RS	Y	N	Y	N	N	N	NA	Y	43%, high
Brandão et al. 2021 [37]	CS	Y	Y	Y	Y	Y	Y	NA	Y	100%, low
Carreras-Presas et al., 2021 [26]	CS	Y	Y	Y	N	Y	Y	NA	Y	85%, low
Chen et al., 2020 [67]	CSS	Y	N	Y	Y	N	N	NA	Y	57%, moderate
Chérif et al., 2020 [38]	CR	Y	Y	Y	Y	Y	N	NA	Y	85%, low
Chiotos et al. 2020 [39]	CS	Y	Y	Y	Y	Y	Y	NA	Y	100%, low
Chiu et al., 2020 [40]	CR	Y	N	Y	Y	Y	Y	NA	Y	85%, low
Ciccarese et al., 2021 [41]	CR	Y	Y	Y	Y	Y	Y	NA	Y	100%, low
Cruz-Tapia et al. 2020 [68]	CS	Y	Y	Y	N	N	N	NA	Y	57%, moderate
De Medeiros et al., 2021 [42]	CR	Y	Y	Y	Y	Y	N	NA	Y	85%, low
Díaz Rodríguez et al., 2020 [43]	CS	Y	N	Y	Y	Y	Y	NA	Y	85%, low
Dima et al., 2020 [44]	CS	Y	Y	Y	Y	Y	N	NA	Y	85%, low
El Kady et al., 2021 [82]	CSS	Y	N	Y	N	N	N	NA	Y	43%, high
Emelyanova et al., 2021 [69]	CR	N	N	Y	Y	Y	N	NA	Y	57%, moderate
Fathi et al. 2021 [45]	CR	Y	N	Y	Y	Y	N	NA	Y	71%, low
Favia et al. 2021 [70]	CSS	Y	N	Y	Y	N	N	NA	Y	57%, moderate
Fernandez-Nieto et al., 2020 [46]	CSS	Y	Y	Y	Y	Y	N	NA	Y	85%, low
Gabusi et al., 2021 [47]	CR	Y	Y	Y	Y	Y	Y	NA	Y	100%, low

Gherlone et al., 2021 [48]	CSS	Y	Y	Y	Y	Y	N	NA	Y	85%, low
Halepas et al., 2021 [71]	CSS	Y	N	Y	Y	N	N	NA	Y	57%, moderate
Hedou et al. 2020 [83]	CSS	Y	N	N	N	N	N	NA	Y	28%, high
Hockova et al., 2021 [21]	CS	Y	Y	Y	Y	Y	Y	Y	Y	100%, low
Horzov et al., 2021 [84]	RS	Y	N	N	N	Y	N	NA	Y	43%, high
Ibarra et al., 2020 [49]	CCS	Y	N	Y	N	Y	Y	NA	Y	71%, low
Jiménez-Cahué et al., 2020 [50]	CS	Y	Y	Y	Y	Y	Y	NA	Y	100%, low
Jones et al. 2020 [51]	CR	Y	Y	Y	Y	Y	N	NA	Y	85%, low
Jiménez-Cahué et al., 2020 [85]	CSS	U	N	Y	Y	N	N	NA	Y	43%, high
Kämmerer et al., 2021 [52]	CR	Y	N	Y	Y	Y	N	NA	Y	71%, low
Katz and Yue, 2021 [86]	OS	Y	N	Y	N	N	N	NA	Y	43%, high
Labè et al., 2020 [72]	CS	Y	N	Y	U	N	Y	NA	Y	57%, moderate
Llamas-Velasco et al., 2020 [53]	CS	Y	Y	Y	Y	N	N	NA	Y	71%, low
Martel and Orgill, 2020 [73]	CS	Y	N	Y	N	Y	N	Y	Y	57%, moderate
Mascitti et al., 2020 [54]	CSS	N	N	Y	Y	Y	Y	NA	Y	71%, low
Mazzotta et al., 2020 [74]	CR	Y	N	Y	Y	N	N	NA	Y	57%, moderate
McGoldrick et al., 2021 [87]	CS	Y	N	N	Y	N	N	NA	Y	43%, high
Marouf et al., 2021 [55]	CCS	Y	Y	Y	Y	Y	Y	NA	Y	100%, low
Nuno-Gonzalez et al. 2021 [88]	CSS	U	N	Y	N	N	N	NA	Y	28%, high
Perrillat et al., 2020 [56]	CS	Y	Y	Y	Y	Y	N	NA	Y	85%, low
Ramires et al., 2021 [57]	CR	Y	Y	Y	Y	Y	Y	NA	Y	100%, low
Ramondetta et al., 2020 [58]	CR	Y	Y	Y	N	Y	N	NA	Y	71%, low
Rekhtman et al., 2021 [59]	CSS	Y	Y	Y	N	Y	N	NA	Y	71%, low
Riad et al., 2020 [89]	CS	U	N	Y	N	N	N	NA	Y	28%, high
Salehi et al., 2020 [75]	CSS	Y	N	N	N	Y	Y	NA	Y	57%, moderate
Shearer et al., 2021 [90]	RS	Y	N	N	N	Y	N	NA	Y	43%, high
Singh et al. 2020 [60]	CS	Y	Y	Y	Y	Y	Y	NA	Y	100%, low
Sinjari et al., 2020 [76]	OS	Y	N	Y	Y	N	N	NA	Y	57%, moderate
Siotos et al., 2020 [77]	CR	Y	Y	Y	N	N	N	NA	Y	57%, moderate
Sleiwah et al., 2020 [78]	RS	Y	Y	N	N	N	N	Y	Y	57%, moderate
Soares et al. 2020 [61]	CR	Y	N	Y	Y	Y	Y	NA	Y	85%, low
Taşkın et al., 2020 [62]	CR	Y	N	Y	Y	Y	Y	NA	Y	85%, low
Taşlıdere et al., 2021 [63]	CR	Y	Y	Y	Y	Y	N	NA	Y	85%, low
Teixeira et al., 2021 [64]	CS	Y	Y	Y	Y	Y	Y	NA	Y	100%, low
Verdoni et al. 2020 [65]	OS	Y	Y	Y	Y	Y	Y	NA	Y	100%, low
Zingarelli et al., 2020 [66]	CR	Y	Y	Y	Y	Y	N	NA	Y	85%, low

Table S2. Certainty assessment and grading of the evidence

Certainty assessment							№ of patients		Effect		Certainty	Importance
**№ of studies**	Study design	Risk of bias	Inconsistency	Indirectness	Imprecision	Other considerations	[intervento]	[confronto]	Relative (95% CI)	Absolute (95% CI)		
Oral mucosal lesions in hospitalized patients with COVID-19 (assessed with: not applicable)												
59	observational studies	serious[a]	serious	not serious	not serious	all plausible residual confounding would reduce the demonstrated effect dose response gradient	19 case reports, 17 case series, 2 case-control studies, 13 cross-sectional studies, 4 obser-vational studies and 4 retrospective studies				⨁⨁◯◯ Low	IMPORTANT

CI: confidence interval. Explanations a. Risk of bias moderate according to Joanna Brigs Institute Appraisal Tool.
